# Supplementary material for: Orexin-A and endocannabinoids are involved in obesity-associated alteration of hippocampal neurogenesis, plasticity, and episodic memory in mice
Source: Nat Commun. 2021 Oct 21;12:6137. doi: 10.1038/s41467-021-26388-4 (PMC8531398; doi:10.1038/s41467-021-26388-4)
Supplement: Supplementary file 1 — Supplementary Information [file 41467_2021_26388_MOESM1_ESM.pdf]

Supplementary Figure 1

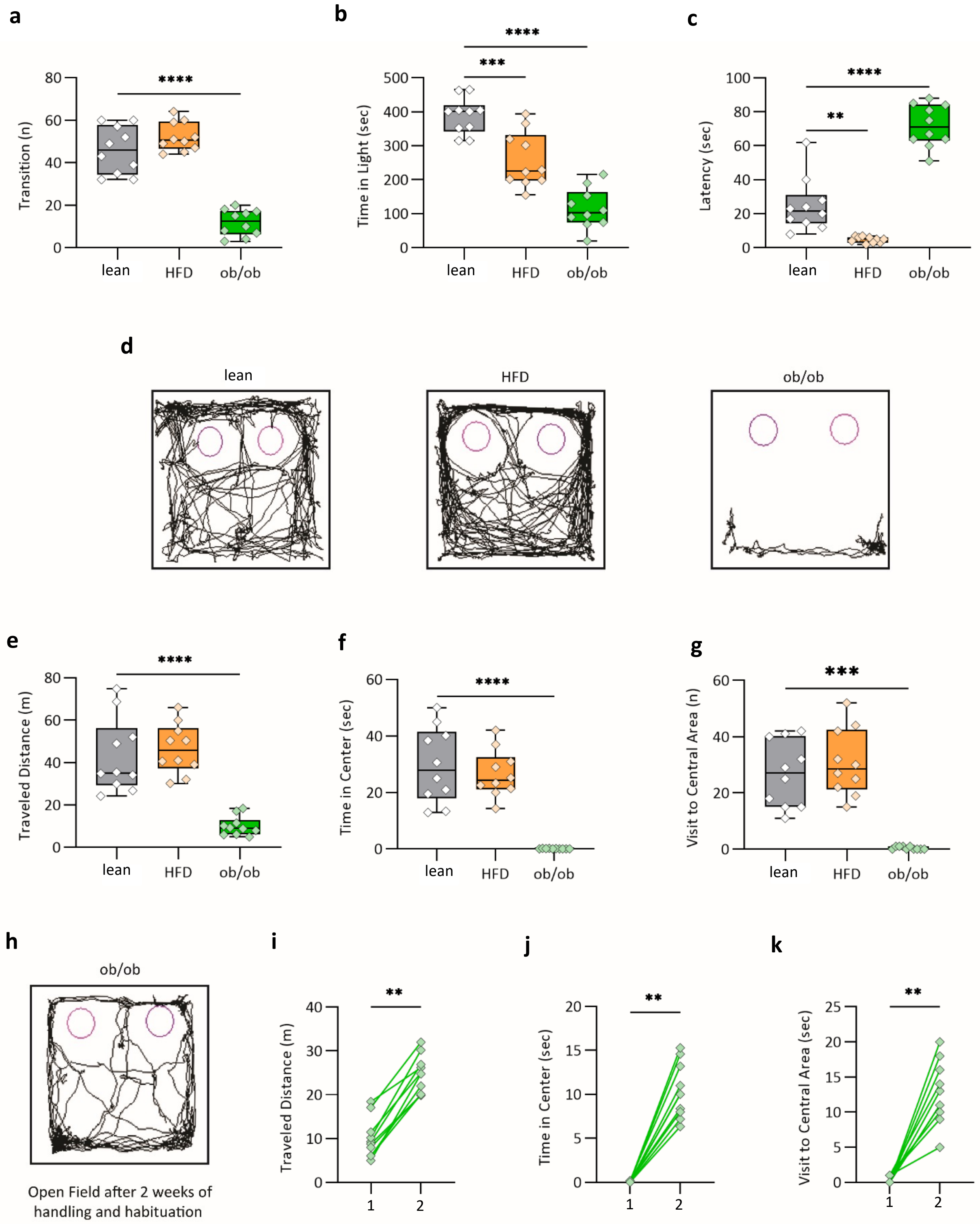

Supplementary Figure 1. Light/Dark box and Open Field in HFD and ob/ob mice. (a-c) Transition, time in light, and latency of entry into the dark compartment during the light/dark box behavioral assay in lean, HFD and ob/ob mice. n=10 mice per group. (a) Transition: lean=45.9±3.5, HFD=52.10±2.1, ob/ob=11.8±1.9, ANOVA test and Bonferroni post hoc test, ***p<0.001, F=67.72. (b) Time in Light: lean=387.9±16.8sec, HFD=258.4±25.6sec, ob/ob=115.1±18.6sec, ANOVA test and Bonferroni post hoc test, ***p<0.001, F=43.3; (c) Latency: lean=24.90±5sec, HFD=4.8±0.5sec, ob/ob=71.90±3.9sec, ANOVA test and Bonferroni post hoc test, ***p<0.001 and *p<0.05, F=87.02. (d) Position tracking of example lean, HFD, and ob/ob mice respectively. (e-g) Traveled distance, visit to central area, and time spent in the center of the area calculated during an open field behavioral assay in lean, HFD, and ob/ob mice. n=10 mice per group. (e) Travelled Distance: lean=43±5.5m, HFD=46.46±3.7m, ob/ob=10.04±1.4m, ANOVA test and Bonferroni post hoc test, **p<0.01, F=23.57. (f) Time in Center: lean=29.6±4.1sec, HFD=26.51±2.6sec, ob/ob=0.4±0.1sec, ANOVA test and Bonferroni post hoc test, ***p<0.001, F=32.34. (g) Visit to Central Area: lean=26.8±3.7, HFD=30.80±3.7 n, ob/ob=0.4±0.1, Kruskal-Wallis test and post hoc Dunn's test, ***p<0.001, Kruskal-Wallis statistic=19.93. (h) Position tracking of a representative ob/ob mouse after 2 weeks of handling and habituation. (i-k) Traveled distance, visit to central area, and time spent in the center of the area calculated before and after the 2 weeks of handling and habituation, n=10 mice per group. (i) Traveled distance, (j) time spent in the center, (k) visit to central area; 1 = traveled distance, time spent in the center or number of visits to the central area calculated during the first open field, 2= traveled distance, time spent in the center or number of visits to the central area calculated after 2 weeks of habituation. Travelled Distance after 2 weeks=24.71±1.3m; Time in Center after 2 week =10.4+0.9sec; Visit to central Area after 2 week = 12.70±1.4sec, Wilcoxon Test, **p<0.01. The box plots elements are: center line, median (Q2); square symbol, mean; box limits, 25th (Q1)-75th (Q3) percentiles; whisker length is determined by the outermost data points. Source data are provided as a Source Data file.

Supplementary Figure 2

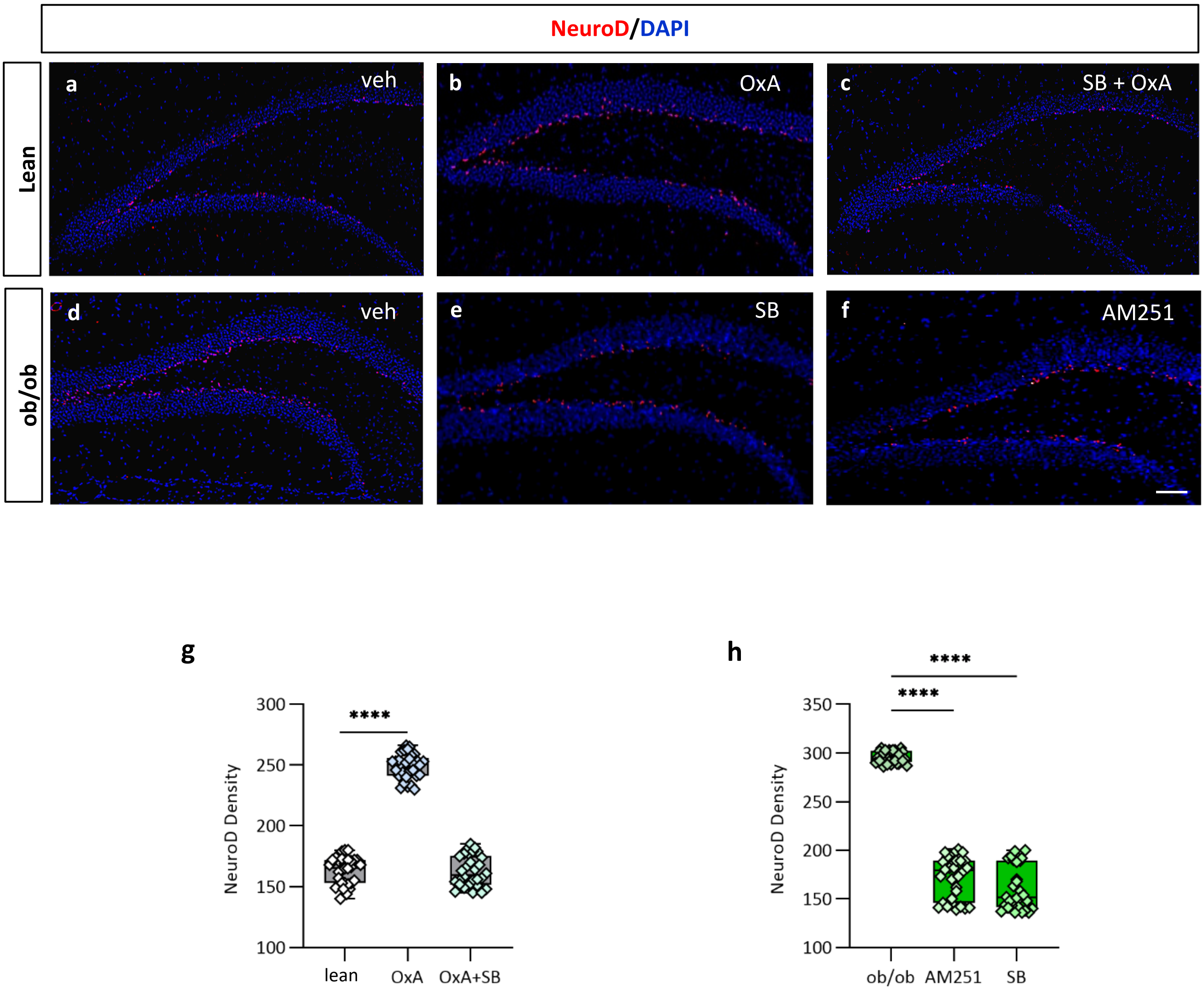

Supplementary Figure 2. **OxA enhances the number of NeuroD-positive neurons via Ox1-R and CB1 receptors.** (a-f) Representative images by confocal microscopy of the DG showing NeuroD-positive newborn neurons (red), in lean mice treated with OxA (280 pmol icv) or OxA+SB and in ob/ob mice treated with AM251 (3mg/Kg) or SB (60mg/Kg, i.p). Scale bar 200µm. (g) Box plots showing the mean number of NeuroD-immunopositive cells in the DG of lean mice injected with vehicle (n=163.1±2.1), or OxA (n=248.8±1.9) or OxA+SB (n=162.2±2.2); n=30 slices in 6 mice per group Kruskal-Wallis test and post hoc Dunn's test, ****$p<0.0001$, Kruskal-Wallis statistic= 60.81. (h) Box plots showing the mean number of NeuroD-immunopositive cells in the DG of ob/ob injected with vehicle (n=295.4±1.1) or AM251 (n=171.6±3.9) or SB (n=161.3±4.2); n=30 slices in 6 mice per group Kruskal-Wallis test and post hoc Dunn's test, ****$p<0.0001$, Kruskal-Wallis statistic= 60.42. The box plots elements are: center line, median (Q2); square symbol, mean; box limits, 25th (Q1)-75th (Q3) percentiles; whisker length is determined by the outermost data points. Immunolabeling was repeated n=3 times independently in different DG sections from n=6 mice per group, with similar results. Source data are provided as a Source Data file.

Supplementary Figure 3

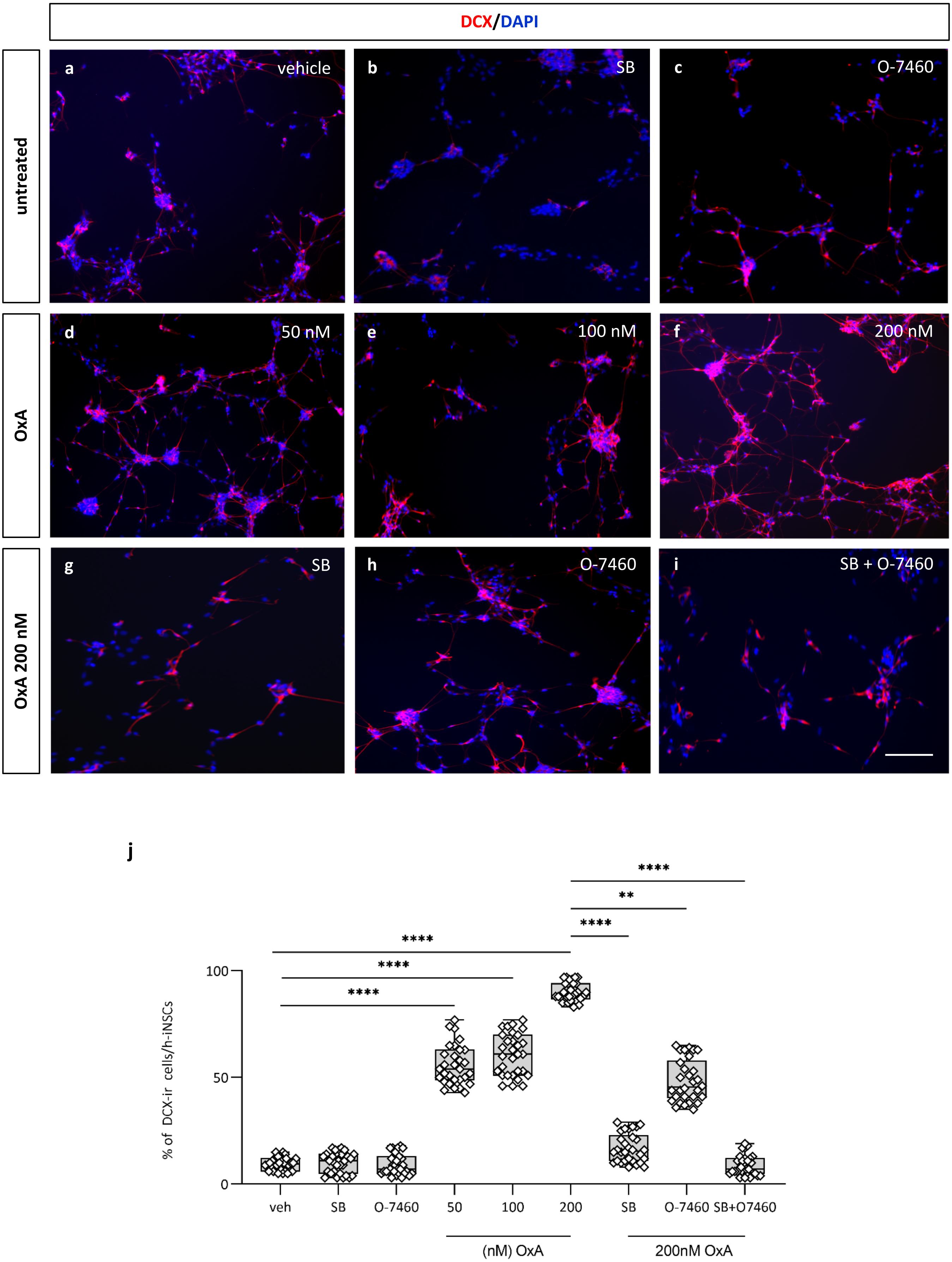

Supplementary Figure 3. OxA stimulates neuronal differentiation of h-iPSCs. (a-i) Confocal images of DCX-ir cell profiles (red) obtained by differentiation of human-induced pluripotent stem cells (iPSCs, Axol #ab0013) labeled with DAPI (blue). Cells were cultured 72h starting from the pre-differentiative cell stage in the appropriate cell medium (vehicle) (a), alone or by adding SB334867 (10μM) (b), O-7460 (10μM) (c) or OxA (50nM, 100nM or 200nM) (d-f), also in combination with SB334867 (10μM) (g), O-7460 (10μM) (h) or SB334867 + O-7460 (i) 15 min before treatment with OxA 200nM. Scale Bar: 100μm. (j) Box plots report the mean of % of DCX-ir cells in different conditions: Veh=9.4% ± 0.55%, SB=10.3% ± 0.86%, O7460=9.1% ± 0.87%, OxA 50nM=55.87% ± 1.7%, OxA 100nM=60.73% ± 1.8%, OxA 200nM=90.13% ± 0.7%, OxA200nM + SB=16.90% ± 1.22%, OxA200nM + O7460=48.37% ± 1.7%, OxA200nM + SB + O7460 = 8.7% ± 0.7%; Kruskal-Wallis test and post hoc Dunn's test, $^{**}p<0.01$, $^{****}p<0.0001$, Kruskal-Wallis statistic=224.2, n=9 since each biological replicate (n=3) has n=3 technical replicates. The box plots elements are: center line, median (Q2); square symbol, mean; box limits, 25th (Q1)-75th (Q3) percentiles; whisker length is determined by the outermost data points. Source data are provided as a Source Data file.

SUPPLEMENTARY TABLE 1

Orexin-A and endocannabinoids are involved in obesity-associated alteration of hippocampal neurogenesis, plasticity, and episodic memory in mice.

A) Box Plot Values and exact P values

Fig.	Box Plot Values	lean	HFD	ob/ob		P value				
1b	Minimum	0.03000	0.000	0.05600		0.1373				
	25% Percentile	0.04750	0.02750	0.07400						
	Median	0.08500	0.07000	0.1250						
	75% Percentile	0.1275	0.1400	0.1600						
	Maximum	0.1700	0.1500	0.1700						
	Range	0.1400	0.1500	0.1140						
1c	Minimum	0.1400	-0.6300	-0.2000		0.0005				
	25% Percentile	0.2450	-0.1400	-0.1850						
	Median	0.3000	0.02500	0.02500						
	75% Percentile	0.3800	0.1700	0.1750						
	Maximum	0.6000	0.2000	0.3000						
	Range	0.4600	0.8300	0.5000						
1d	Minimum	0.1000	-0.3300	-1.000		0.0003				
	25% Percentile	0.1775	0.007500	-0.4250						
	Median	0.2500	0.08500	0.000						
	75% Percentile	0.3050	0.2075	0.08400						
	Maximum	0.5000	0.2500	0.1000						
	Range	0.4000	0.5800	1.100						
1e	Minimum	-0.1818	-0.1417	-0.2400		0.6857				
	25% Percentile	-0.1210	-0.02676	-0.07500						
	Median	0.04601	0.1000	0.04927						
	75% Percentile	0.08836	0.1287	0.1144						
	Maximum	0.2500	0.3000	0.2286						
	Range	0.4318	0.4417	0.4686						
1f	Minimum	0.1370	0.1200	0.1900		0.6329				
	25% Percentile	0.1854	0.3300	0.3225						
	Median	0.4824	0.5450	0.6000						
	75% Percentile	0.6200	0.6725	0.7400						
	Maximum	0.8100	0.9000	0.8000						
	Range	0.6730	0.7800	0.6100						
1g	Minimum	0.07692	0.1100	0.1100		0.6012				
	25% Percentile	0.1795	0.2250	0.2475						
	Median	0.3961	0.5800	0.4200						
	75% Percentile	0.6421	0.6775	0.6225						

	**Maximum**	0.8000	0.7700	0.7000						
	**Range**	0.7231	0.6600	0.5900						
**3a**	**Minimum**	56.00	45.00	43.00						
	**25% Percentile**	67.50	52.00	45.75						
	**Median**	76.50	60.00	53.50						
	**75% Percentile**	81.25	67.25	64.50						
	**Maximum**	91.00	75.00	73.00						
	**Range**	35.00	30.00	30.00						
**3b**	**Minimum**	145.0	226.0	235.0						
	**25% Percentile**	164.3	235.3	249.5						
	**Median**	174.0	248.5	264.0						
	**75% Percentile**	182.5	268.0	275.3						
	**Maximum**	193.0	285.0	284.0						
	**Range**	48.00	59.00	49.00						
**3c**	**Minimum**	140.0	280.0	286.0						
	**25% Percentile**	154.3	289.5	290.8						
	**Median**	163.0	300.5	293.5						
	**75% Percentile**	177.0	307.0	302.0						
	**Maximum**	187.0	320.0	305.0						
	**Range**	47.00	40.00	19.00						
**3d**	**Minimum**	12.00	23.00	20.00						
	**25% Percentile**	15.00	28.75	24.75						
	**Median**	18.50	34.50	29.50						
	**75% Percentile**	23.00	39.25	34.25						
	**Maximum**	28.00	43.00	40.00						
	**Range**	16.00	20.00	20.00						
		**lean**			**HFD**			**ob/ob**		
		**gcld**	**pd**	**md**	**gcld**	**pd**	**md**	**gcld**	**pd**	**md**
**3k**	**Minimum**	2.000	2.000	2.000	2.000	5.000	2.000	2.000	6.00	3.000
	**25% Percentile**	2.000	2.750	5.000	2.000	6.000	8.750	3.750	7.00	7.000
	**Median**	3.000	3.500	6.000	3.000	7.000	11.00	4.000	9.00	9.000
	**75% Percentile**	4.000	4.000	8.000	4.000	9.000	14.25	5.000	12.0	12.25
	**Maximum**	5.000	5.000	9.000	5.000	10.00	16.00	9.000	16.0	16.00
	**Range**	3.000	3.000	7.000	3.000	5.000	14.00	7.000	10.0	13.00
		**lean**	**HFD**	**Obob**		**p value**				
**4d**	**Minimum**	87.40		74.00		0.5362				
	**25% Percentile**	105.4		75.76						
	**Median**	115.4		137.0						
	**75% Percentile**	139.8		173.6						
	**Maximum**	160.6		258.0						
	**Range**	73.17		184.0						

**4h**	**Minimum**	218.4	79.24	67.49		0.0022				
	**25% Percentile**	218.8	79.68	80.49						
	**Median**	256.0	94.11	93.49						
	**75% Percentile**	281.7	108.2	132.2						
	**Maximum**	306.1	108.6	147.8						
	**Range**	87.71	29.34	80.35						
		**lean**		**HFD**	**ob/ob**					
		**Veh**	**OxA**	**Veh**	**Veh**	**Lep**				
**5a**	**Minimum**	4.098	9.035	9.000	9.020	4.041				
	**25% Percentile**	4.170	9.249	9.400	10.08	4.080				
	**Median**	5.077	10.17	10.09	11.07	5.048				
	**75% Percentile**	5.162	10.24	11.80	12.05	5.878				
	**Maximum**	6.150	12.15	12.60	12.15	7.063				
	**Range**	2.052	3.111	3.600	3.125	3.022				
		**lean**	**100nM OXA**	**200nM OXA**	**200nM OXA +SB**		**P value**			
**5d**	**Minimum**	87.40	90.97	57.85	74.91		0.0229			
	**25% Percentile**	105.4	96.89	69.75	90.49					
	**Median**	115.4	114.7	87.60	118.1					
	**75% Percentile**	139.8	139.8	112.5	130.3					
	**Maximum**	160.6	148.3	118.7	148.8					
	**Range**	87.40	90.97	57.85	74.91					
		**CB1**			**OxA**			**CB1/OxA**		
		**Lean**	**HFD**	**obob**	**Lean**	**HFD**	**obob**	**Lean**	**HFD**	**obob**
**5h**	**Minimum**	18.00	17.00	20.00	3.000	25.00	27.00	0.00	0.00	0.00
	**25% Percentile**	24.75	28.50	34.75	9.000	33.25	33.75	0.00	0.00	0.00
	**Median**	36.50	44.50	48.50	15.50	44.00	39.00	1.00	1.00	1.00
	**75% Percentile**	52.25	58.25	58.50	19.00	48.00	48.75	2.00	2.25	3.00
	**Maximum**	61.00	66.00	67.00	28.00	55.00	56.00	2.00	3.00	3.00
	**Range**	43.00	49.00	47.00	25.00	30.00	29.00	2.00	3.00	3.00
		**lean**	**HFD**	**Ob/ob**						
**5i**	**Minimum**	6.000	23.00	21.00						
	**25% Percentile**	10.75	37.25	46.00						
	**Median**	13.50	53.50	62.50						
	**75% Percentile**	19.50	81.00	75.50						
	**Maximum**	25.00	86.00	89.00						
	**Range**	19.00	63.00	68.00						
**5j**	**Minimum**	60.00	68.00	61.00						
	**25% Percentile**	65.75	71.00	66.00						
	**Median**	76.50	76.00	75.50						
	**75% Percentile**	81.00	85.75	85.00						
	**Maximum**	85.00	95.00	96.00						

	Range	25.00	27.00	35.00						
		lean			HFD		Ob/ob			
		Veh	OxA	SB+OxA	Veh	SB	Veh	SB		
6a	Minimum	1.560	9.580	2.760	17.08	4.060	16.58	6.990		
	25% Percentile	2.213	11.31	3.140	18.70	7.145	16.92	7.163		
	Median	2.500	12.91	4.015	20.05	7.865	18.30	8.335		
	75% Percentile	4.863	18.10	5.148	21.67	9.115	20.57	8.758		
	Maximum	5.130	20.38	6.630	22.16	9.880	23.16	9.590		
	Range	3.570	10.80	3.870	5.080	5.820	6.580	2.600		
		lean	AM251	O-7460	ACEA	P value				
7b	Minimum	87.40	84.50	76.46	56.26	0.0149				
	25% Percentile	105.4	101.3	82.91	70.20					
	Median	115.4	121.6	126.6	87.50					
	75% Percentile	139.8	182.2	145.8	95.75					
	Maximum	160.6	190.2	157.8	113.0					
	Range	87.40	84.50	76.46	56.26					
		HFD	SB	AM251		P value				
7d	Minimum	79.24	125.3	100.2		0.0048				
	25% Percentile	79.68	141.2	100.7						
	Median	94.11	191.9	109.8						
	75% Percentile	108.2	253.9	117.8						
	Maximum	108.6	273.6	117.9						
	Range	79.24	125.3	100.2						
		obob	SB	AM251		P value				
7f	Minimum	67.49	165.6	165.6		0.0029				
	25% Percentile	80.49	167.1	167.1						
	Median	93.49	175.3	175.3						
	75% Percentile	132.2	179.6	179.6						
	Maximum	147.8	179.9	179.9						
	Range	80.35	14.24	14.24						
7k						P value				
						0.0013				
		Lean	HFD	obob		P value				
8b	Minimum	0.01000	0.01000	0.000		0.2788				
	25% Percentile	0.05825	0.04250	0.04000						
	Median	0.1029	0.06500	0.1188						
	75% Percentile	0.1775	0.1125	0.1775						
	Maximum	0.2900	0.1400	0.2300						
	Range	0.2800	0.1300	0.2300						

8c	**Minimum**	0.1000	0.1500	0.2000		0.6845				
	**25% Percentile**	0.1625	0.1675	0.3200						
	**Median**	0.3788	0.3200	0.3650						
	**75% Percentile**	0.7150	0.4625	0.4541						
	**Maximum**	1.300	0.9300	1.000						
	**Range**	1.200	0.7800	0.8000						
8d	**Minimum**	0.000	-0.1700	-0.3300		0.0013				
	**25% Percentile**	0.1225	-0.1100	-0.3300						
	**Median**	0.3175	0.01000	0.000						
	**75% Percentile**	0.4700	0.1550	0.1325						
	**Maximum**	0.5600	0.3000	0.2700						
	**Range**	0.5600	0.4700	0.6000						
**SUPPLEMENTARY FIGURES**										
		**Lean**	**HFD**	**obob**						
1a	**Minimum**	32.00	3.000	44.00						
	**25% Percentile**	34.25	6.250	46.50						
	**Median**	46.00	12.50	50.50						
	**75% Percentile**	57.75	17.25	59.25						
	**Maximum**	60.00	20.00	64.00						
	**Range**	28.00	17.00	20.00						
1b	**Minimum**	315.0	20.00	155.0						
	**25% Percentile**	342.8	73.75	197.8						
	**Median**	401.0	103.3	226.0						
	**75% Percentile**	419.5	163.0	331.5						
	**Maximum**	465.0	216.0	394.0						
	**Range**	150.0	196.0	239.0						
1c	**Minimum**	8.000	51.00	2.000						
	**25% Percentile**	14.25	63.00	3.000						
	**Median**	21.50	*71.00*	5.000						
	**75% Percentile**	31.00	84.25	6.250						
	**Maximum**	62.00	88.00	7.000						
	**Range**	54.00	37.00	5.000						
1e	**Minimum**	24.20	5.000	19.88						
	**25% Percentile**	29.20	6.075	20.08						
	**Median**	35.15	9.035	24.90						
	**75% Percentile**	56.18	12.90	27.73						
	**Maximum**	74.80	18.43	32.00						
	**Range**	50.60	13.43	12.12						

1f	Minimum	11.00	0.000	15.00						
	25% Percentile	15.00	0.000	21.25						
	Median	27.00	0.000	28.50						
	75% Percentile	40.25	1.000	42.50						
	Maximum	42.00	1.000	52.00						
	Range	31.00	1.000	37.00						
	Minimum	11.00	0.000	15.00						
1g	Minimum	12.90	0.000	14.33						
	25% Percentile	17.98	0.000	21.09						
	Median	27.81	0.000	24.29						
	75% Percentile	41.40	0.1600	32.50						
	Maximum	50.10	0.2000	42.00						
	Range	37.20	0.2000	27.67						
		P value								
1i		0.0020								
1j		0.0020								
1k		0.0020								
		Lean	HFD	obob						
2g	Minimum	140.0	230.0	145.0						
	25% Percentile	153.0	241.0	151.8						
	Median	165.5	250.5	159.5						
	75% Percentile	172.0	255.8	175.5						
	Maximum	180.0	266.0	185.0						
	Range	40.00	36.00	40.00						
2h	Minimum	286.0	136.0	139.0						
	25% Percentile	290.8	141.8	145.8						
	Median	293.5	151.5	179.0						
	75% Percentile	302.0	189.3	189.0						
	Maximum	305.0	200.0	201.0						
	Range	19.00	64.00	62.00						
		veh	SB	O7460	50	100	200	SB	O7460	SB+O7460
3j	Minimum	5.000	3.000	3.000	43.00	46.00	83.00	8.000	35.0	3.000
	25% Percentile	6.000	5.000	5.000	48.75	51.00	86.75	11.00	40.5	5.000
	Median	9.500	11.00	7.000	54.00	61.00	88.50	15.50	45.5	7.000
	75% Percentile	12.00	14.00	13.00	63.00	70.00	94.00	22.75	57.7	12.00
	Maximum	15.00	17.00	18.00	77.00	77.00	97.00	29.00	65.0	19.00
	Range	10.00	14.00	15.00	34.00	31.00	14.00	21.00	30.0	16.00

B) STATISTICAL VALUES OF FIG 2:

Figure 2b

DAY1: Diet $F_{1,14}$=0.021; p=0.87

DAYS 2-4: Diet $F_{1,28}$=0.02; p=0.88; Latency $F_{2,28}$=24.37; p<0.0001; Diet x Latency $F_{2,28}$=1.69; p=0.2

DAYS 5-6: Diet $F_{1,14}$=4.9; p=0.04; Latency $F_{1,14}$=1.097; p=0.31; Diet x Latency $F_{1,14}$=1.097; p=0.31

Figure 2c

DAY1: Diet $F_{1,28}$=0.01; p=0.89

DAYS 2-4: Diet $F_{1,28}$=0.002; p=0.99; Path length $F_{2,28}$=22.89; p<0.0001; Diet x Path length $F_{2,28}$=1.21; p=0.3

DAYS 5-6: Diet $F_{1,14}$=4.38; p=0.05; Path length $F_{1,14}$=0.91; p=0.35; Diet x Path length $F_{1,14}$=1.7; p=0.2

Figure 2d: Diet $F_{1,14}$=1.74; p=0.24; Time $F_{1,14}$=56.9; p<0.0001; Diet x Time $F_{2,28}$=1.49; p=0.24

Figure 2f: Diet $F_{1,14}$=4.82; p=0.04

Figure 2g: Diet $F_{1,14}$=5.55; p=0.03

Figure 2h: Diet $F_{1,14}$=4.88; p=0.04
