# Supplementary material for: Orexin-A and endocannabinoids are involved in obesity-associated alteration of hippocampal neurogenesis, plasticity, and episodic memory in mice
Source: Nat Commun. 2021 Oct 21;12:6137. doi: 10.1038/s41467-021-26388-4 (PMC8531398; doi:10.1038/s41467-021-26388-4)
Supplement: Supplementary file 3 — Reporting Summary [file 41467_2021_26388_MOESM3_ESM.pdf]

nature portfolio

Corresponding author(s): Vincenzo Di Marzo, Luigia Cristino

Last updated by author(s): Sep 9, 2021

Reporting Summary

Nature Portfolio wishes to improve the reproducibility of the work that we publish. This form provides structure for consistency and transparency in reporting. For further information on Nature Portfolio policies, see our Editorial Policies and the Editorial Policy Checklist.

Statistics

For all statistical analyses, confirm that the following items are present in the figure legend, table legend, main text, or Methods section.

n/a	Confirmed	
☐	☒	The exact sample size (*n*) for each experimental group/condition, given as a discrete number and unit of measurement
☐	☒	A statement on whether measurements were taken from distinct samples or whether the same sample was measured repeatedly
☐	☒	The statistical test(s) used AND whether they are one- or two-sided<br>*Only common tests should be described solely by name; describe more complex techniques in the Methods section.*
☒	☐	A description of all covariates tested
☐	☒	A description of any assumptions or corrections, such as tests of normality and adjustment for multiple comparisons
☐	☒	A full description of the statistical parameters including central tendency (e.g. means) or other basic estimates (e.g. regression coefficient) AND variation (e.g. standard deviation) or associated estimates of uncertainty (e.g. confidence intervals)
☐	☒	For null hypothesis testing, the test statistic (e.g. *F*, *t*, *r*) with confidence intervals, effect sizes, degrees of freedom and *P* value noted<br>*Give P values as exact values whenever suitable.*
☒	☐	For Bayesian analysis, information on the choice of priors and Markov chain Monte Carlo settings
☒	☐	For hierarchical and complex designs, identification of the appropriate level for tests and full reporting of outcomes
☒	☐	Estimates of effect sizes (e.g. Cohen's *d*, Pearson's *r*), indicating how they were calculated

Our web collection on statistics for biologists contains articles on many of the points above.

Software and code

Policy information about availability of computer code

Data collection	Immunohistochemistry: Nikon Eclipse Ti2 (Nikon, Florence, Italy) . ELISA test: Multiskan GO; Thermo Scientific. 2AG levels: labsolution shimadzu; Electron Microscopy: FEI Tecnai G2 Spirit TWIN; Immunocytochemistry: Leica DMI6000 Fluorescence Microscope; ELISA Test: Multiskan GO (Thermo Scientific, Waltham, MA); 2-AG Levels: Spectrometry Mass Shimadzu ; Western Blotting Analysis: Chemi-Doc station; In vitro Electrophysiology: Leica DM6000 FS microscope equipped with a WAT-902H Ultimate camera, MultiClamp 700B and Digidata 1440A (Axon Instruments, Molecular Devices, Sunnyvale, CA, USA) and the pClamp 10.4 software (Molecular Devices), isolated pulse stimulator AM 2100 (A-M Systems, Carlsborg, WA). In vivo Electrophysiology: Win LTP 2.30, Digitimer ltd. model DS3 ; Behaviour: stopwatch (Silva, Sweden), automated behavioral tracking system (Smart v3.0, Panlab Harvard Apparatus), video camera (PANASONIC WV-BP330), video-tracking system (ANY-MAZE 7.08, Stoelting, USA).
Data analysis	Immunohistochemistry: NIS-Elements C and Adobe Photoshop 6.01, LAS-X Measurement (Leica) ; Immunocytochemistry:Metamoph Imaging Software (Leica MetaMorph AF); 2-AG Levels: LabSolution Software Shimadzu; Western Blotting Analysis: Quantity-one software (Bio-Rad, Milan, Italy); In vitro Electrophysiology: Clampfit 11.1 (Molecular Devices); In vivo Electrophysiology: Win LTP 2.30; Behaviour: automated behavioral tracking system (Smart v3.0, Panlab Harvard Apparatus), video-tracking system (ANY-MAZE 7.08, Stoelting, USA). Statistical analysis: GraphPad Prism 8 (GraphPad Software, USA)

For manuscripts utilizing custom algorithms or software that are central to the research but not yet described in published literature, software must be made available to editors and reviewers. We strongly encourage code deposition in a community repository (e.g. GitHub). See the Nature Portfolio guidelines for submitting code & software for further information.

Data

Policy information about availability of data

All manuscripts must include a data availability statement. This statement should provide the following information, where applicable:

- Accession codes, unique identifiers, or web links for publicly available datasets
- A description of any restrictions on data availability
- For clinical datasets or third party data, please ensure that the statement adheres to our policy

All data generated or analysed during this study are included in this published article (and its supplementary information files).

Field-specific reporting

Please select the one below that is the best fit for your research. If you are not sure, read the appropriate sections before making your selection.

- [x] Life sciences
- [ ] Behavioural & social sciences
- [ ] Ecological, evolutionary & environmental sciences

For a reference copy of the document with all sections, see nature.com/documents/nr-reporting-summary-flat.pdf

Life sciences study design

All studies must disclose on these points even when the disclosure is negative.

Sample size	No sample size calculations were performed. The minimal sample-size useful to detect a statistical significattive difference between groups was adopted. Sample size was chosen based on previous experience and standards in the field (Barbieri et al. 2018; Cristino et al, 2013; De Risi et al, 2021)
Data exclusions	Data were not excluded in the analysis
Replication	All experiments were performed at least in quadruplicate. For immunohistochemical experiments, at least nine individual slices were analyzed. For immunocytochemical experiment at least n=3 biological replicates were performed, each having at least n=3 tecnical replicates. For in vitro electrophysiology maximun three slices per mouse were recorded. All attempts of replication were successful and gave similar results.
Randomization	All experiments in this work were performed using animals and animal-derived samples, each group were selected randomly. The experimental subset involving iPSCs Axol ab0013 cells was analyzed equally with no sub-sampling and thus, there was no requirement of randomization.
Blinding	For data collection and analysis, the investigators were blinded.

Reporting for specific materials, systems and methods

We require information from authors about some types of materials, experimental systems and methods used in many studies. Here, indicate whether each material, system or method listed is relevant to your study. If you are not sure if a list item applies to your research, read the appropriate section before selecting a response.

Materials & experimental systems

n/a	Involved in the study	
[ ]	[x]	Antibodies
[ ]	[x]	Eukaryotic cell lines
[x]	[ ]	Palaeontology and archaeology
[ ]	[x]	Animals and other organisms
[x]	[ ]	Human research participants
[x]	[ ]	Clinical data
[x]	[ ]	Dual use research of concern

Methods

n/a	Involved in the study	
[x]	[ ]	ChIP-seq
[x]	[ ]	Flow cytometry
[x]	[ ]	MRI-based neuroimaging

Antibodies

Antibodies used	Goat anti-OxA (Santa Cruz, SC-8070; for IHC 1:100 or for CLEM 1:50); goat anti-Neuro-D (Santa Cruz, SC-1084; 1:100); guinea pig-anti VGluT1 (Synaptic System, 135 304, 1:200); goat anti-Ox1R (Santa Cruz, sc-8072, 1:50); rabbit anti-CB1 receptor anti C terminus 461-472, (Abcam, ab23703, for IHC 1:200 or for CLEM 1:50); goat anti-CB1 receptor antibody (Santa Cruz; sc-10068; 1:100); rabbit DAGL alpha Antibody C-terminal (Affinity Biosciences, DF13509, 1:50); rabbit anti-NMDAR2B (Abcam, cat. no. ab65783, 1:1000); goat anti-NMDAR2B (Novus Biologicals, NB100-41097, 1:200); rabbit anti-DCX (Abcam, ab18732; 1:100) or mouse anti-DCX (1:100; Santa Cruz, sc-271390); mouse anti-PSD95 (1:100; SYSY, cat. no. 124011); monoclonal anti-tubulin (cat. T8203 Sigma-Aldrich; Milan, Italy).

	Immunofluorescence was revealed by specific Alexa secondary donkey anti-IgGs (Invitrogen, ThermoFisher Scientific, France): alexa-350 donkey anti-goat (A21081, 1:50 or 1:100); alexa-350 donkey anti-rabbit (A10039, 1:100); alexa-488 donkey anti-rabbit (A21206, 1:50); alexa-488 donkey anti-mouse (A21202, 1:50); alexa-488 goat anti-guinea pig (A11073, 1:100); alexa-594 donkey anti-mouse (A21203, 1:50); alexa-594 donkey anti-goat (A11058, 1:50).
Validation	This antibody anti-OxA (Santa Cruz, SC-8070) was validated in our previous work (Cristino et al 2013, PNAS) and many other works (Slater, PG. et al. 2016; Hunt, N.J. et al. 2015 see Santa Cruz website).<br>anti-Neuro-D (Santa Cruz, SC-1084) and anti-Ox-1R antibody (Santa Cruz, SC8072 1:200) antibodies have been validated by Santa Cruz by demonstrating immunoblotting on whole cell lysates and inhibition of this signal with an immunizing peptide (see website for other citations).<br>VGluT1 (Synaptic System, 135 304) antibodies has been validated by Synaptic System by demonstrating immunoblotting on rat brain extract and in rat, mouse and guinea pig brain tissues by immunohistochemistry.<br>rabbit anti-CB1R (anti C terminus 461-472, Abcam, ab23703 and ab40860) antibodies has been validated by Abcam by demonstrating immunoblotting on human cerebellum extract and in rat brain tissue by immunohistochemistry.<br>DAGL alpha Antibody - C-terminal (Affinity Biosciences, DF13509) has been validated by Affinity Bioscinces on rat and mouse brain tissue.<br>anti-NMDAR2B (Abcam, cat. no. ab65783) antibody has been validated by Abcam in SKNSH cells and PC12 cells and many other works (Unsicker et al 2021; Wheatley et al 2019; Guo et al 2029).<br>rabbit-anti-DCX (Abcam, ab207175) has been validate by abcam in rat adult hippocampal brain.<br>mouse-anti-DCX (Santa Cruz, cat. no. sc-271390) has been validated by Santa Cruz on mouse hippocampus.<br>mouse anti PSD95 (SYSY, cat. no. 124011) was tested and used in many other work (Mannara et al 2020, Grabrucker et al 2021, Li J et al 2020), see SYSY datasheet.<br>anti-tubulin (cat. T8203, Sigma-Aldrich) was tested using differe cellular lysates Neuro2A, HeLa, HEK, Rat brain, NRK and others, see datasheet

Eukaryotic cell lines

Policy information about cell lines

Cell line source(s)	iPSCs, Axol ab0013
Authentication	The iPSCs, Axol ab0013 were identified according to the Axol's Product Catalogue and datasheet
Mycoplasma contamination	the cell lined tested was negative to mycoplasma contamination
Commonly misidentified lines (See ICLAC register)	none

Animals and other organisms

Policy information about studies involving animals; ARRIVE guidelines recommended for reporting animal research

Laboratory animals	12-13-weeks old mice ob/ob, JAX mouse strain B6. Cg-Lepob/J and proopiomelanocortin (POMC)-eGFP mice (JAX mouse strain, C57BL/6J-Tg(Pomc-EGFP)1Low/J) were used.
Wild animals	n/a
Field-collected samples	n/a
Ethics oversight	The study has been performed according to the ARRIVE Guidelines to improve the reporting of bioscience research using laboratory animals. Experiments were performed following the European Union animal welfare guidelines [European Communities Council Directive of September 22, 2010 (2010/63/EU)] and the Italian Decree n.26/2014, authorization n. 152/2020-PR and 589/2018.

Note that full information on the approval of the study protocol must also be provided in the manuscript.
